# Cardiac magnetic resonance imaging versus computed tomography to guide transcatheter aortic valve replacement: study protocol for a randomized trial (TAVR-CMR)

**DOI:** 10.1186/s13063-022-06638-6

**Published:** 2022-09-02

**Authors:** Gert Klug, Sebastian Reinstadler, Felix Troger, Magdalena Holzknecht, Martin Reindl, Christina Tiller, Ivan Lechner, Priscilla Fink, Mathias Pamminger, Christian Kremser, Hanno Ulmer, Axel Bauer, Bernhard Metzler, Agnes Mayr

**Affiliations:** 1 University Clinic of Internal Medicine III, Cardiology and Angiology, Medical University of Innsbruck, Anichstraße 35, 6020 Innsbruck, Austria; 2 University Clinic of Radiology, Medical University of Innsbruck, Innsbruck, Austria; 3 Department for Medical Statistics, Informatics and Health Economy, Medical University of Innsbruck, Innsbruck, Austria

**Keywords:** Transcatheter aortic valve replacement, Cardiac magnetic resonance, Computed tomography, Kidney injury

## Abstract

**Background:**

The standard procedure for the planning of transcatheter aortic valve replacement (TAVR) is the combination of echocardiography, coronary angiography, and cardiovascular computed tomography (TAVR-CT) for the exact determination of the aortic valve dimensions, valve size, and implantation route. However, up to 80% of the patients undergoing TAVR suffer from chronic renal insufficiency. Alternatives to reduce the need for iodinated contrast agents are desirable. Cardiac magnetic resonance (CMR) imaging recently has emerged as such an alternative. Therefore, we aim to investigate, for the first time, the non-inferiority of TAVR-CMR to TAVR-CT regarding efficacy and safety end-points.

**Methods:**

This is a prospective, randomized, open-label trial. It is planned to include 250 patients with symptomatic severe aortic stenosis scheduled for TAVR based on a local heart-team decision. Patients will be randomized in a 1:1 fashion to receive a predefined TAVR-CMR protocol or to receive a standard TAVR-CT protocol within 2 weeks after inclusion. Follow-up will be performed at hospital discharge after TAVR and after 1 and 2 years. The primary efficacy outcome is device implantation success at discharge. The secondary endpoints are a combined safety endpoint and a combined clinical efficacy endpoint at baseline and at 1 and 2 years, as well as a comparison of imaging procedure related variables. Endpoint definitions are based on the updated 2012 VARC-2 consensus document.

**Discussion:**

TAVR-CMR might be an alternative to TAVR-CT for planning a TAVR procedure. If proven to be effective and safe, a broader application of TAVR-CMR might reduce the incidence of acute kidney injury after TAVR and thus improve outcomes.

**Trial registration:**

The trial is registered at ClinicalTrials.gov (NCT03831087). The results will be disseminated at scientific meetings and publication in peer-reviewed journals.

## Administrative information

*Note*: the numbers in curly brackets in this protocol refer to SPIRIT checklist item numbers. The order of the items has been modified to group similar items (see http://www.equator-network.org/reporting-guidelines/spirit-2013-statement-defining-standard-protocol-items-for-clinical-trials/).

**Table:** 

Title {1}	Cardiac Magnetic Resonance Imaging Versus Computed Tomography to Guide Transcatheter Aortic Valve Replacement: A Randomized Trial (TAVR-CMR)
Trial registration {2a and 2b}.	ClinicalTrials.gov (NCT03831087)
Protocol version {3}	29^th^ of July 2019. Version 4.0
Funding {4}	Applied.
Author details {5a}	Klug Gert, MD^1^ Reinstadler Sebastian, MD, PhD^1^ Troger Felix, MD^1,^^2^ Holzknecht Magdalena, MD^1^ Reindl Martin, MD, PhD^1^ Tiller Christina, MD, PhD^1^ Lechner Ivan, MD^1^ Fink Priscilla, MD^1^ Pamminger Mathias, MD^2^ Kremser Christian, PhD^2^ Ulmer Hanno, PhD^3^ Bauer Axel, MD^1^ Metzler Bernhard, MD, MSc, FESC^2^ Mayr Agnes, MD^2^ ^1^University Clinic of Internal Medicine III, Cardiology and Angiology, Medical University of Innsbruck, Austria ^2^ University Clinic of Radiology, Medical University of Innsbruck, Austria ^3^ Department for Medical Statistics, Informatics and Health Economy, Medical University of Innsbruck, Austria
Name and contact information for the trial sponsor {5b}	Prof. Axel Bauer, MD Medical University of Innsbruck Anichstrasse 35 A-6020 Innsbruck Austria
Role of sponsor {5c}	In this investigator-initiated trial the role of the sponsor is limited to ensuring the infrastructure for the trial. The principal investigator takes over the role of the sponsor regarding study design; collection, management, analysis, and interpretation of data; writing of the report; and the decision to submit the report for publication. The sponsor has no authority over any of these activities.

## Introduction

### Background and rationale {6a}

Severe aortic stenosis is defined as an aortic valve area (AVA) of ≤ 1.0cm^2^ and a mean transaortic valve gradient of ≤ 40 mmHg [1]. The treatment of symptomatic patients with aortic valve replacement (AVR) is an accepted and well validated [2–6] class I B indication in ESC and ACC/AHA guidelines [1, 7]. Recent randomized controlled trials have proven the benefits of transcatheter aortic valve replacement (TAVR) in patients with symptomatic severe aortic stenosis and intermediate to very-high surgical risk [8–10]. Accordingly, the most recent guidelines recommend TAVR in patients with severe symptomatic aortic stenosis and a predicted life expectancy > 12 months with a class I B if surgical risk is prohibitive and a class IIa C indication if patients are at high surgical risk [7]. The decision for TAVR must be based on the agreement of the “Heart Valve Team” [1]. Since its introduction, more than 350,000 patients have undergone TAVR and its utilization rapidly increases. In some countries more than 50% of isolated AVRs are performed via transcatheter techniques [11].

The standard procedure for the evaluation of patients for TAVR is the combination of transthoracic and transesophageal echocardiography (TTE and TEE), coronary angiography, and cardiovascular computed tomography (TAVR-CT) for the exact determination of the aortic valve dimensions, valve size, and implantation route [12]. This approach is based on the use of ionated contrast agents in three different occasions (angiography, CT, and implantation). However, up to 80% of the patients undergoing TAVR suffer from chronic renal insufficiency [13]. Reduced renal function and the amount of contrast agent used for CT have been linked to contrast induced acute kidney injury [14]. Acute kidney injury occurs in 12% of TAVR procedures and is an independent predictor of in-hospital mortality (odds ratio (OR): 4.14, CI 1.42–12.13) [15]. In Austria, 1426 (~ 17/100,000/year) TAVR procedures were performed in 2019 [16] and 24,386 (~ 29/100,000/year) TAVRs were performed in Germany in 2019, respectively [17]. Based on this data more than 17,000 patients with impaired renal function undergo TAVR each year in Austria and Germany. It can be estimated that nigh on 3000 of these patients will suffer from periprocedural acute kidney injury. As recent data have shown that 3% need in-hospital renal replacement therapy and 2% need chronic renal replacement therapy 6 months after TAVR, this sums up to 750 and 500 patients per year [18, 19]. Therefore, alternatives to reduce the need for iodinated contrast agents are desirable [20, 21]. Cardiac magnetic resonance (CMR) imaging recently has emerged as such an alternative [22, 23].

La Manna et al. investigated 49 patients that were planned to undergo TAVR with TEE and cine steady-state free precession (SSFP) CMR and found a moderate correlation of both methods (*r*^2^: 0.58) regarding aortic annulus size and CMR showed a significant trend to overestimation (TEE 21.4 ± 1.9 mm CMR: 23.7 ± 2.2 mm) [22]. However, annulus sizing was only performed in one dimension in this study. Jabbour et al. investigated 202 consecutive patients, which were scheduled for TAVR with CMR, CT, and TEE and demonstrated a small bias between CMR and CT (0.39 mm) but a large bias between CMR or CT and TEE (4.52 mm and 4.1 mm) for the largest aortic valve (AV) annulus diameter. The authors conclude that CMR and CT are both reliable methods for the sizing of the AV annulus before TAVR [24]. However, in this study, no assessment of peripheral arteries and vascular access routes has been performed. To study the impact of calcification on the agreement of CMR and CT for the sizing of the aortic annulus, Tsang et al. performed ex vivo scans on calcium containing aortic rings and demonstrated that CMR and CT both have a very good correlation with known ring diameters and near zero biases. CMR, however, had smaller limits of agreement than CT (− 0.94 to 1.30 versus − 1.08 to 1.79). Furthermore, an increase in calcification leads to an increase in measurement variability for TEE and CT [25]. Koos et al. compared CMR and CT in 58 patients which were evaluated for TAVR and found no relevant difference between CMR and CT regarding annulus diameter (23.4 ± 1.8 mm and 23.6 ± 1.8 mm, *p* = 0.86), left coronary ostial height (13.2 ± 1.8 mm and 13.3 ± 1.7 mm, *p* = 0.86), and the length of the left coronary leaflet (12.3 ± 1.4 mm and 12.3 ± 1.4 mm, *p* = 0.81). The authors conclude however, “in absence of gold standard a randomized prospective study may give insight into the imaging modality resulting in best procedural outcome” [26]. Recently, Ruile at el. investigated the aortic roots of 69 patients who underwent pre-TAVR CT with 3D non-contrast fast low angle shot (FLASH) CMR and found good agreement of both methods regarding the measurement of aortic annulus area (intraclass correlation 0.961) as well as the distance to the right and left coronary artery (intraclass correlation 0.894 and 0.797) [27]. Promising results have been achieved by Renker et al. in 10 healthy volunteers when comparing a non-contrast self-navigated 3D sequence to a standard 2D-SSFP CMR protocol to study aortic dimensions form the aortic root to the iliac arteries. This study promises a completely contrast-free CMR protocol for the planning of valve size and access routes before TAVR. Although currently the transfemoral approach is predominantly in use, CMR could also be a promising tool enabling to plan TAVR via alternate access routes [28]. However, further feasibility studies in patients with aortic stenosis and calcified peripheral vessels are needed [28]. Novel approaches even utilize real-time CMR for the guidance of TAVR and report easy and effective deployment with the use of the CoreValve and a minimally modified catheter system, which opens the field for a further reduction in contrast and radiation dose during the course of a TAVR procedure [29, 30]. Furthermore, the use of CMR in patients with degenerated bioprosthetic aortic valves before TAVR was shown to be feasible [31].

We have previously presented a fully CMR-guided approach to TAVR planning including aortic root sizing, determination of ostial and leaflet distances, and the evaluation of peripheral vascular access routes [32]. Moreover, we showed for the first time the feasibility of a completely non-contrast enhanced CMR protocol and presented a prototype, navigator-free “whole-heart” CMR angiography for TAVR planning [33, 34]. According to the presented data, CMR is considered as a safe alternative to CT and TEE in the planning of TAVR procedures by leading experts in the field [12, 35, 36]. However, no randomized trial has shown the non-inferiority of CMR to CT in terms of efficacy and safety.

### Objectives {7}

The primary objective of the present study is (a) to prove the non-inferiority of TAVR-CMR compared to TAVR-CT to plan TAVR according to clinical efficacy, defined as implantation success based on the VARC-2 criteria [37]. Secondary objectives are to prove the non-inferiority of TAVR-CMR compared to TAVR-CT to plan TAVR with regard to (b) a combined safety endpoint, based on the VARC-2 criteria [37]. Furthermore, (c) an extended clinical efficacy and safety follow-up for the non-inferiority of TAVR-CMR versus CT after 2 years as well as (d) assess imaging procedure related variables and (e) to study the accuracy of TAVR-CMR and TAVR-CT for the evaluation of patients undergoing TAVR compared to TAVR-TEE measurements.

### Trial design {8}

**Fig. 1:**
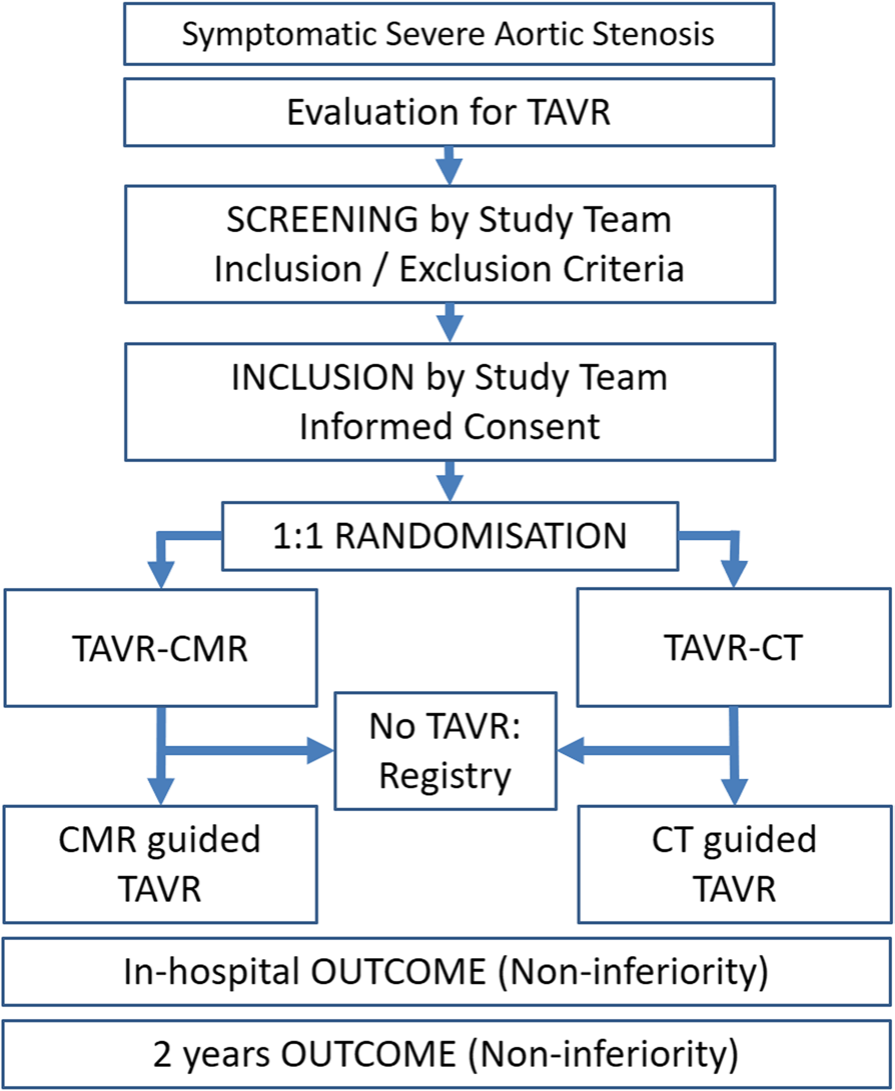
Study design flow chart. *TAVR*, transcatheter aortic valve replacement; *CMR*, *cardiovascular magnetic resonance*; *CT*, *computed tomography*

The present trial is a prospective, randomized, open-label trial to prove the non-inferiority of TAVR-CMR compared to TAVR-CT to guide TAVR. The final analysis will include 226 consecutive patients scheduled for TAVR. According to previous studies, approximately 45% of the patients are female [9]. Therefore, to guarantee gender equity, we aim to enroll at least 80 female patients. Patients will be randomized in a 1:1 fashion to receive a predefined TAVR-CMR protocol or to receive a standard contrast-enhanced TAVR-CT protocol within 2 weeks after inclusion. Inclusion and exclusion criteria are presented below. A detailed study flowchart is presented by Fig. 1. Follow-up for the primary objective will be performed before discharge after TAVR. Extended analysis and clinical follow-up is planned after 2 years.

### Patient involvement

Our present study included no involvement of public or patient representatives concerning the design of the protocol.

## Methods

### Study setting {9}

This project will be carried out as a single-center study located at the University Clinic for Internal Medicine III (Cardiology) and the University Clinic of Radiology at Innsbruck Medical University (Radiology Core Lab). Moreover, the infrastructure available at Innsbruck Medical University including the Department of Medical Statistics, Informatics and Health Economics as well as the Clinical Trial Center will assist the conduct of the study and warranting the successful accomplishment of the project.

### Eligibility criteria {10}

Patients aged from 19 to 99 years with a decision for TAVR for symptomatic severe aortic stenosis according to the decision of the local “heart valve team”. Screening will be performed during the “heart valve team” meetings, which are conducted once a week. Eligible patients will then be asked for study participation and checked for the following inclusion and exclusion criteria.

Inclusion criteria:

1. Signed informed consent
2. Severe aortic stenosis according to recent guidelines (aortic valve area ≤ 1.0cm^2^ or aortic valve index ≤ 0.6 cm^2^/m^2^) [1]
3. Typical symptoms of severe aortic stenosis like shortness of breath, angina, or syncope
4. Decision for TAVR according to the local “heart valve team” including cardiologists and cardiac surgeons

Exclusion criteria:

1. Contraindications to perform CMR
2. Contraindications to perform CT
3. Contraindications for TAVR or reduced life expectancy < 1 year.
4. Known hypersensitivity to CMR or CT contrast agents
5. Killip class ≥ 3
6. Childbearing potential or inability to exclude pregnancy
7. Inability to understand and follow study-related instructions
8. Severe renal insufficiency requiring renal replacement therapy
9. Severe hepatic insufficiency (Child–Pugh class B or C)
10. Post organ transplantation
11. Participation in another clinical study

Withdrawal criteria:

1. Patients request without giving reasons
2. Administrative decision by the investigator
3. Pregnancy
4. Significant protocol deviation
5. Subject noncompliance
6. Adverse event
7. Other safety concern of the investigator or sponsor
8. Lost to follow-up

### Who will take informed consent? {26a}

After the identification of the individual patient, he will be asked by a senior physician of the study team to give informed consent to the participation in the study.

### Additional consent provisions for collection and use of participant data and biological specimens {26b}

In this study, the patient consents to the performance of all study procedures including the study intervention, blood samples, and further investigations. No storage or biobanking of biological specimens is planned.

### Interventions

#### Explanation for the choice of comparators {6b}

Currently, only few publications investigated the role of CMR to guide TAVR planning. So far, only small trials have been published and they investigate only one aspect (aortic root dimensions or peripheral arteries) of the whole planning procedure [32, 33]. What is more, these studies have not been performed in a randomized fashion.

#### Intervention description {11a}

##### Cardiovascular magnetic resonance (CMR)

All CMR examinations will be performed with a 1.5 T clinical MR imaging unit (AVANTO_fit; Siemens, Erlangen, Germany). The MR protocol consists of a Navigator-gated free breathing 3D “whole-heart” coronary magnetic resonance angiography (MRA), axial 2D true fast imaging with steady-state free precession (true-FISP) during free breathing covering whole body trunk, and a coronal 3D fast low-angle shot (FLASH) Gd-MRA.

- Navigator-gated free breathing 3D “whole-heart” coronary MRA
  Planning of the whole-heart acquisition includes the placement of a cubic field of view at the level of the bulbus aortae as identified at axial, coronal, and parasagittal localizers. The acquisition method is both cardiac triggered and respiratory gated. For an accurate respiratory gating, real-time position of the diaphragm is monitored by the use of navigator echos. A line of signal is reconstructed from each navigator echo and displayed as a trace. The boundary between the low signal intensity in the lung and the relatively high signal intensity in the liver creates an edge that can easily be detected and used as a gating signal, which accurately reflects the diaphragm position and is used to determine whether the data is accepted or rejected. The accuracy of this method allows narrow gating windows (3.5 mm) to be set for high resolution applications. Following MR parameters will be used for this “whole-heart technique”: repetition time (TR) [ms]/echo time (TE) [ms]: 356/1.57; field of view (FOV): 350 × 262 × 58 mm; matrix: 148 × 256 × 72; acquired voxel size: 1.36 × 1.36 × 0.8 mm; flip angle: 90°; and receiver bandwidth: 592 Hz/pixel.
- 3D fast low-angle shot (FLASH) Gd-MRA

Coronal breath-hold fast low-angle shot (FLASH) 3D-MRA will be acquired before and after the intravenous administration of 0.2 mmol/kg of Gd-DO3A-butriol (Gadovist™, Schering, Berlin, Germany) at 2 ml/s, followed by 20 ml of saline flush, administered using an automatic injector (Spectris Injection System, Medrad, Pittsburgh, USA). To define the time delay between contrast injection and image acquisition, we use a semi-automatic synchronization protocol based on the visual inspection of the contrast bolus arrival in the left ventricle (care-bolus). Following MR parameters will be used: TR/TE: 3/1 ms; flip angle: 30°; one excitation; 112 partitions; slice thickness: 1.25 mm; pixel size, 1.3 mm × 1.3 mm × 1.3 mm; acquisition time: 12 s.

##### Computed tomography (CT)

All CT examinations will be performed on a 128-slice dual-source CT (128 mm × 0.6 mm detector collimation, 0.28 s gantry rotation time) and high-pitch factor (3.2; Somatom Definition Flash, Siemens Healthcare, Forchheim, Germany). Prospective electrocardiographic synchronization will be applied, triggered into the diastolic phase for the heart. An injected bolus of 70 to 110 mL of nonionic iodine contrast agent will be applied with 370 mg/mL iodine concentration (Iopromide, Ultravist 370, Bayer Schering Pharma, Berlin, Germany), using an automatic injector at a flow rate of 5 mL/s, followed by 40 mL saline solution. Contrast agent volume for each patient will be calculated by scan time and body weight. Patients will be placed supine with arms overhead. The scan length range from supraaortic branches to the groin.

##### CMR and CT data analysis

Both, an experienced cardiologist and radiologist rate CMR and CT data in a blinded manner. Measurements will be performed at reconstructed 3D “whole-heart” coronary MRA and aortic CTA by rotating the segmented aortic root and left ventricular chamber:

- Rating of aortic valve morphology (bicuspid vs tricuspid)
- Measurements of the aortic annulus at the 3 connection points (“hinge points“) of the aortic valve cusp insertion (“deepest coronary sinus points”)
- Measurements of coronary ostia heights of the left and right coronary artery (= distance from the annulus)

At true-FISP and contrast-enhanced MRA as well as CTA:

- Complete standardized peripheral TAVR-planning including aortic and iliac artery sizing

The choice of prosthesis size for both Edwards Sapien and CoreValve as well as the suitability of the TAVR procedure via femoroiliac access is made for each imaging modality separately, based on manufacturer’s recommendations. Sapien 23 mm is chosen for an annulus perimeter range of 60–70.5 mm, the 26 mm valve for a perimeter range of 70.5–80 mm, and the 29 mm valve for a perimeter range of 80–90 mm, whereas perimeter ranges (69–72 mm) and (78.5–81.5 mm) is considered to fit for both, lower and larger valve size. CoreValve 23 mm is chosen for an annulus perimeter range of 56.5–62.8 mm, the 26 mm valve for a perimeter range of 62.8–72.3 mm, the 29 mm valve for a perimeter range of 72.3–84.8 mm, and CoreValve 31 mm for a perimeter range of 81.7–91.1, whereas perimeter range (81.7–91.1 mm) was considered to fit for both, CoreValve 29 mm and CoreValve 31 mm.

#### Criteria for discontinuing or modifying allocated interventions {11b}

In this study, following withdrawal criteria apply:

1. Patients request without giving reasons
2. Administrative decision by the investigator
3. Pregnancy
4. Significant protocol deviation
5. Subject noncompliance
6. Adverse event
7. Other safety concern of the investigator or sponsor
8. Lost to follow-up

The sponsor is authorized to discontinue the study due to relevant medical/administrative causes. The reasons for the discontinuation of the study have to be documented in detail. Patients, who are still under treatment at the time of discontinuation, have to be examined for a final investigation, which will be documented in the CRF. If the investigator has any ethical qualms concerning the continuation of the study, this has to be immediately reported to the sponsor.

The sponsor is authorized to discontinue the study, if.

- The recruitment rate of patients is not sufficient
- Serious, non-resolvable problems of quality of the collected data evolve
- Unpredictable circumstances in the particular study centers appear, which do not allow a continuation of the study
- New scientific findings during the study’s run-time do not allow a continuation of the study

The study administration is able to decide about the discontinuation of the study in agreement with the sponsor, the Data Safety Monitoring Board, or the protocol committee.

The study must be closed on completion. As far as possible, premature closure should occur after mutual consultation. Depending on local legislation, it may be necessary to inform the ethics committees and the regulatory authorities when the study site is closed.

Upon closure of the entire study or a single center, all study materials (completed, partially completed and blank CRFs, study medication, etc.) must be returned to the sponsor and disposed or archived as directed by the sponsor.

#### Strategies to improve adherence to interventions {11c} and relevant concomitant care permitted or prohibited during the trial {11d}, adverse event reporting and harms {22}

Cross-over between allocated groups because of clinical reasons should be minimized and will be documented. Non-contrast CT scans to assess calcium load if CMR is not sufficient should also be minimized especially by discussing special cases within the TAVR team including TAVR operators and radiologists. Patients included in the study are informed that they should not participate in another experimental study during the study period. Furthermore, no restrictions are made regarding clinical care of the patients. Adverse events will be immediately reported to the sponsor.

### Outcomes {12}

Follow-up will be conducted at discharge as well as 2 years after TAVR. Follow-up study investigations are summarized in Table 1. End-point definitions will be based on the updated 2012 VARC-2 consensus document [37].

**Table 1:**
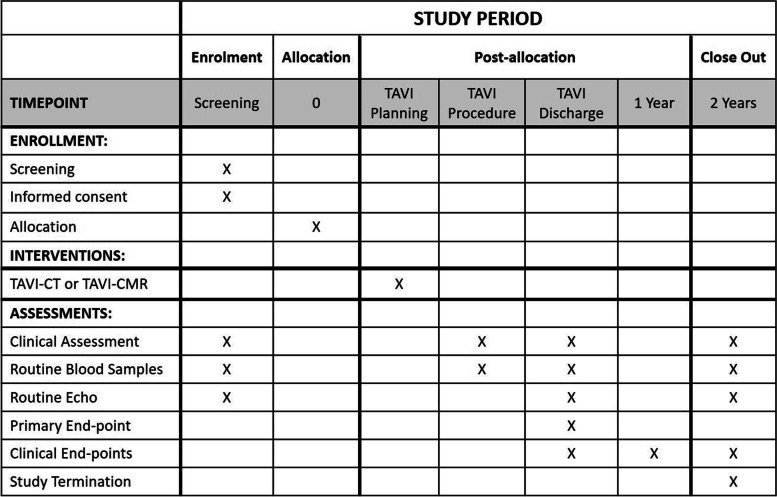
Individual study time-table

*TAVR* Transcatheter aortic valve replacement, *CMR* Cardiovascular magnetic resonance, *CT* Computed tomography

#### Primary endpoint

The primary outcome will be a composite clinical efficacy end-point related to implantation success at hospital discharge, based on the VARC-2 criteria [37]

- Absence of procedural mortality
- Correct positioning of the prosthetic valve
- Intended performance of the prosthetic valve (mean aortic valve gradient < 20 mmHg and no valve regurgitation > mild)

#### Secondary endpoints

Secondary end-points consist of clinical efficacy and safety end-points at discharge and 2 years.

*Combined safety end-point:*

- All-cause mortality
- All strokes
- Life-threatening bleeding
- Acute kidney injury—stage 2 or 3 (including renal replacement therapy)
- Coronary artery obstruction requiring intervention
- Major vascular complications
- Valve-related dysfunction requiring repeat procedure

*Combined clinical efficacy end-point:*

- All-cause mortality
- All stroke
- Hospitalization for aortic valve related symptoms or heart failure
- NYHA class III or IV
- Prosthetic valve related dysfunction (mean gradient > 20 mmHg or regurgitation > mild)

Furthermore, the two imaging strategies will be compared regarding imaging procedure related variables:

- Percentage of interpretable scans and scoring of image quality
- Percentage of patients with need for an alternative imaging method
- Duration of imaging and patient comfort
- Cumulative radiation dose during TAVR planning
- Cumulative amount of contrast agent used for TAVR planning
- Imaging related safety parameters as contrast extravasation, allergic reactions, et cetera.

Finally, biomarker levels at baseline as well as at follow-up visits will be investigated.

### Participant timeline {13} and provisions for post-trial care {30}

The time-table for the individual participant is presented in Table 1. After individual completion of the study period, patients are advised to seek regular follow-up examinations at their referring physician. A patient insurance covers cost for compensation of harm.

### Sample size {14} and recruitment {15}

Sample size estimation for the primary endpoint is based on previous data indicating a rate of 4% of early deaths, 4% of valve regurgitation > mild, and 0.4% of reinterventions after TAVR [10]. Therefore, we estimate an implantation success rate of 92% in both study groups. For a non-inferiority margin of 9%, hence 113 patients per group should be included to achieve a statistical power of 80%. A drop-out rate of 10% is assumed. Therefore, it is planned to include a total of 250 patients in the trial.

## Assignment of interventions: allocation

### Sequence generation {16a}, concealment mechanism {16b}, implementation{16c}

Patients will undergo randomization after informed consent is given. Randomization will be performed with the randomization module of the web-based database program REDCap (Research Electronic Data Capture, REDCap, Vanderbilt University 2016). The allocation sequence is based on computer-generated random numbers without stratification. The allocation table is generated in a separate file uploaded to the REDCap randomization module before the database is going to the production status.

## Assignment of interventions: blinding

### Who will be blinded {17a}, procedure for unblinding if needed {17b}

The randomizing physicians are blinded to the allocation table. Due to the nature of the study, the assignment to the study intervention (diagnostic test) and the image interpretation will be performed open-label. Furthermore, TAVR operators are not blinded. Study personnel responsible for the evaluation of end-points will be blinded regarding the allocated intervention. Unblinding procedures are not necessary due to the open-label design of the study.

## Data collection and management

### Plans for assessment and collection of outcomes {18a} and plans to promote participant retention and complete follow-up {18b}, methods to handle non-adherence and missing data {20c}

Patient data will be pseudonymized for study purposes. All patient data will be assessed on a paper-based CRF. Variables will be entered from clinical available source data (patient record) or via direct entry based on the information given by patients or the clinical examination. For the purpose of electronic data processing, the data will then be transferred into a database (Research Electronic Data Capture, REDCap, Vanderbilt University 2016).

If a patient does not return for a scheduled visit, every effort will be made to contact the patient. In any circumstance, every effort will be made to document patient outcome, if possible. If a patient decides to withdraw from the study, he/she will be contacted in order to obtain information about the reason for discontinuation if the patient agrees. The date and reason for the withdrawal will be recorded in the case report form (CRF). The study is registered at www.clinicaltrials.gov (NCT03831087).

### Data management {19}, methods for additional analyses {20b}, confidentiality {27}, interim analyses {21b}, auditing trial conduct {23}

Principal investigator and co-investigators will monitor observational and medical record data throughout the duration of the study and will operate independently from the funder. The principal investigator will be responsible for all aspects of the trial. All data files will be stored in a locked file cabinet inside our institution. Only the staff listed in the application will have access to the files and at no time will data files be shared with collaborators outside the institution. Patient medical record numbers will be assigned a study ID number in a master key, and study IDs will be used on all research documents. Only the principal investigator, co-investigators, and data manager will have access to the master key. We assure that any publications and presentations of the data will not allow for the identification of patients, hospitals, or physicians. Currently, there are no audits, additional or interim analyses planned.

### Composition of the coordinating center and trial steering committee {5d}, composition of the data monitoring committee, its role, and reporting structure {21a}

The current study will be coordinated by the principal investigator and the steering committee at the Medical University Innsbruck. The principal investigator will be responsible for the process of the study, the completeness of the datasets, consent forms, and correct randomization and report of adverse events.

### Protocol amendments {25}

All relevant changes and modifications to the protocol will be communicated to the contributing investigators, ethics committees, registries, and, if affected, to the trial participants.

### Ancillary and post-trial care {30}

A patient insurance covers cost for compensation of harm. After the study period, patients will receive standard of care in the outpatient setting.

### Dissemination policy {31a}, availability of data and materials {29}

If required, all participants will be informed about their respective findings by mail and can make an inquiry about their results at any time.

In the further course, it is planned to publish our results at national and international conferences as well as in a cardiovascular or radiologic journal. All co-authors of the respective work must meet eligibility criteria, i.e., each one must have significantly contributed to its content. It is planned to grant access to the full protocol on reasonable request.

### Author’s contribution {31b}

GK acts as principal investigator and is, together with SR, involved in conceptualization and study design. FT, MH, MR, CT, IL, and PF are mostly responsible for patient identification and recruitment. MP, CK, and AM are mostly involved in radiological and physical issues concerning study design. HU is the primary contact person for statistical questions and evaluations. Lastly, AB is the head of the Department of Internal Medicine III (Cardiology and Angiology) at the Medical University of Innsbruck and therefore the representative of the sponsor, and BM acts as the head of the respective study group.

### Consent for publication {32}

All study participants give written informed consent concerning study participation and publication of the respective data.

### Plans for collection, laboratory evaluation, and storage of biological specimens for genetic or molecular analysis in this trial/future use {33}

Not applicable, as there are no biological specimens collected as part of the study.

## Statistical methods {20a}

Statistical analysis will be performed with SPSS 26.0. (IBM, Armonk, NY, USA) for Windows.

Statistical analysis will be supervised by an independent statistician from the Department of Medical Statistics, Informatics and Health Economics (Innsbruck Medical University). Normally distributed continuous variables will be presented as mean ± standard deviation, not normally distributed continuous variables as median with corresponding interquartile range (IQR). Categorical variables will be displayed as the number and percentage of patients. Pearson or Spearman correlations will be calculated as appropriate. *χ*^2^-test will be used to compare categorical variables between groups. ANOVA with Bonferroni post hoc testing (if ND) and Mann–Whitney-*U* (if not ND) will be used to determine differences in continuous variables between groups. Time-to-event is defined as time from the TAVR procedure to the date of the first end-point. Kaplan–Meier survival curves will be determined and log-rank comparison will be used between groups. Uni- and multivariate Cox-regression analysis will be used to estimate hazard ratios for all left ventricular variables. Factors with univariate association with the primary end-points with significance < 0.1 will be entered in multivariate analysis. For all data a 2-tailed *p*-value of *p* < 0.05 will be considered to indicate statistical significance. No interim analysis is planned.

## Discussion

This research is highly significant, as a broader usage of CMR in the planning of TAVR might have an immense impact on the incidence of postinterventional kidney injury and might furthermore improve clinical outcome. What is more, TAVR-CMR also allows to plan TAVR via alternate routes, such as the subclavian or axillary artery, if a femoral approach is not possible. Thus, TAVR-CMR could represent a reasonable alternative to the current routine standard of TAVR-CT. With increasing age, aortic stenosis is a major burden for affected patients and, thus, represents a huge challenge for Western health care systems. With growing postoperative risk, patients are more likely to benefit from TAVR; however, especially these patients are at higher risk of suffering periinterventional kidney failure due to a high cumulative dose of contrast agent [13]. This patient population seems to be the main target group to profit from the alternative use of TAVR-CMR. What is more, CMR appeared to show higher accuracy than CT and 3D-echocardiography in assessing aortic annulus dimensions ex vivo as well as in vivo, while CT seemingly tends to underestimate annular size [25]. Due to the potential prevention of postinterventional kidney injury, CMR can also be expected to reduce costs albeit being more expensive upfront. Furthermore, chronic kidney disease and consequential renal replacement therapy have a massive impact on quality of life in terms of physical, functional, metabolic, social, and mental conditions [38], which again adds to treatment costs.

Anyway, we do acknowledge that our study bears some limitations. Firstly, TAVR-CMR is still not widely available and for now can be performed primarily in tertiary-care centers. Additionally, CT surpasses MRI especially in the characterization of aortic and valvular calcification, which indeed is a common concomitant feature of AV stenosis. Nevertheless, it can be assumed that neither vascular nor valvular calcifications will have a major impact on the planning of TAVR via CMR; according to Tsang et al., CMR and CT have a very good agreement in sizing the annular ring diameters independently of calcification grade [25]. Lastly, in patients with claustrophobia or other contraindications for MRI, TAVR-CMR can hardly be performed.

## Trial status

The trial protocol has been approved by Ethics Committee of Medical University Innsbruck. The study initiation was held in July 2019. Current study protocol version number 4.0 is basis for this manuscript and written in accordance with the Standard Protocol Items: Recommendations for Interventional Trials (SPIRIT) Statement.

## Data Availability

The datasets used and/or analyzed during the current study are available from the corresponding author on reasonable request.

## Abbreviations

3D: Three dimensional
ACC: American College of Cardiology
AE: Adverse event
AHA: American Heart Association
AV: Aortic valve
AVA: Aortic valve area
AVR: Aortic valve replacement
CMR: Cardiovascular magnetic resonance
CRF: Case Report Form
CT: Computed tomography
EC: Ethics Committee
ESC: European Society of Cardiology
MRA: Magnetic resonance angiography
MRI: Magnetic resonance imaging
SAE: Serious adverse event
TAVR: Transcatheter aortic valve replacement
TEE: Transesophageal echocardiography
TTE: Transthoracic echocardiography
VARC: Valve Academic Research Consortium
